# Supplementary material for: Sublimation Study of Six 5-Substituted-1,10-Phenanthrolines by Knudsen Effusion Mass Loss and Solution Calorimetry
Source: Entropy (Basel). 2022 Jan 27;24(2):192. doi: 10.3390/e24020192 (PMC8871135; doi:10.3390/e24020192)
Supplement: Supplementary file 1 [file entropy-24-00192-s001.zip › entropy-1555399-supplementary.pdf]

entropy

Supplementary Material

Sublimation study of six 5-substituted-1,10-phenanthrolines. Validation of Knudsen Effusion Mass Loss results from solution calorimetry

Bruno Brunetti [1], Andrea Ciccioli [2], Andrea Lapi [2,3], Aleksey V. Buzyurov [4], Ruslan N. Nagrimanov[4], Mikhail A. Varfolomeev [4,*] and Stefano Vecchio Ciprioti [5,*]

[1] Istituto per lo Studio dei Materiali Nanostrutturati, Consiglio Nazionale delle Ricerche, Dipartimento di Chimica, Sapienza Università di Roma, P.le A. Moro 5, 00185 Rome, Italy;
[2] Dipartimento di Chimica, Sapienza Università di Roma, P.le A. Moro 5, 00185 Rome, Italy,
[3] Istituto per i Sistemi Biologici (ISB-CNR), Sede Secondaria di Roma-Meccanismi di Reazione, c/o Dipartimento di Chimica, Universita` degli Studi di Roma "La Sapienza", P.le A. Moro 5, I-00185 Rome, Italy;
[4] Kazan Federal University, Kremlevskaja Str. 18, 420008 Kazan, Russian Federation; vma.ksu@gmail.com
[5] Dipartimento di Sceinze di Base ed Applicate per l'Ingegneria (S.B.A.I.), Sapienza University of Rome,
Via del Castro Laurenziano 7, Building RM017, I-00161 Rome, Italy; stefano.vecchio@uniroma1.it
* Correspondence: vma.ksu@gmail.com (M.A.V.); stefano.vecchio@uniroma1.it (S.V.C.)

Table S1. Experimental enthalpies of solution of the 5-Cl and 5-CH_3-1,10-phenanthrolines in benzene measured in this work at 298.15 K and 0.1MPa[a].

Compound	Sample mass[b]/mg	Molality[c]/ mmol·kg^{-1}	$\Delta_{soln}H_m^{Aj/S}$ [d] kJ·mol^{-1}
5-Cl-1,10-phenanthroline	31.08	1.65	19.34
	35.26	1.88	19.25
	33.05	3.41	19.64
	33.51	3.66	19.41
	34.95	5.27	19.32
	63.66	7.04	19.37
		$\Delta_{soln}H_m^{Aj/S}$ = (19.39 ± 0.11) kJ·mol^{-1} [e]	
5-CH_3-1,10-phenanthroline	59.17	3.48	18.13
	55.80	3.28	18.33
	31.21	5.31	18.11
	50.91	6.27	18.50
	30.87	7.13	18.62
		$\Delta_{soln}H_m^{Aj/S}$ = (18.34 ± 0.20) kJ·mol^{-1} [e]	

[a]Standard uncertainties u are $u(T)$ = 0.01 K, $u(p)$ = 5 kPa.
[b] Mass of solute sample which was added in each dissolution experiment.
[c] Molality of solute in solution after experiments. Standard uncertainties u are u(b) = 0.08 mmol·kg^{-1}.
[d] Enthalpy of solution of each experiment.
[e] Average enthalpy of solution. Uncertainties correspond to expanded uncertainties of the mean U (0.95 level of confidence. k≈2).

Synthesis of Materials

5-Chloro-1,10-phenanthroline was purchased (Sigma-Aldrich, > 98 %) and used as received. 1,10-phenanthroline-5,6-epoxide, precursor for the synthesis of 5-methoxy-1,10-phenanthroline and 5-cyano-1,10-phenanthroline, was prepared from reaction of 1,10-phenanthroline and hypochlorite according to literature procedure [10].

5-Methoxy-1,10-phenanthroline was synthesized from 1,10-phenanthroline-5,6-epoxide following a procedure previously reported in the literature [10]. A solution of 1,10-phenanthroline-5,6-epoxide (2.0 g, 10.2 mmol) and sodium methoxide (50 mg, 0.93 mmol) in methanol (300 mL) was stirred overnight at room temperature. The solvent was removed by rotary evaporator and the solid residue was dissolved in methylene chloride (10 mL) and filtered. The filtrate was concentrated by rotary evaporator and the compound purified by recrystallization from methylene chloride/hexane (2:5) affording pure 5,6-dihydro-5-hydroxy-6-methoxy-1,10-phenanthroline (1.28 g, 5.6 mmol, 55 %) used in the next step.

NaH (1.54 g, 64 mmol) was added in three portions at 30 min intervals to a stirred solution of 5,6-dihydro-5-hydroxy-6-methoxy-1,10-phenanthroline (1.28 g, 5.6 mmol) in dry dioxane (130 mL) at 70 °C. The reaction mixture was stirred overnight at 80 °C. After cooling to room temperature, the solvent was removed by rotary evaporator and the residue was dissolved in cold water (400 mL). The mixture was extracted with chloroform (3 × 400 mL) and the collected organic fractions, dried over anhydrous Na_2SO_4, were evaporated. The solid thus obtained was purified by chromatography (silica gel, chloroform) affording pure (mass fraction purity > 0.999 %, measured by an Agilent 6890N GC apparatus equipped with a 30 m × 0.25 mm 5% phenylsilicone column) 5-Methoxy-1,10-phenanthroline as a white solid (0.71 g, 3.36 mmol, 60 %). ^{1}H NMR (300 MHz, $CDCl_3$) δ (ppm): 4.10 (s, 3H), 6.95 (s, 1H), 7.58 (dd, 1H, J = 7.91 Hz, J' = 4.43 Hz), 7.65 (dd, 1H, J = 8.13 Hz, J' = 4.29 Hz), 8.16 (d, 1H, J = 7.95 Hz), 8.67 (d, 1H, J = 8.07

Hz), 9.06 (d, 1H, J = 3.75 Hz), 9.21 (d, 1H, J = 3.45 Hz). The ^{1}H NMR spectrum is consistent with that reported in the literature [10].

5-Cyano-1,10-phenanthroline was synthesized from 1,10-phenanthroline-5,6-epoxide following a procedure previously reported in the literature [10]. A solution of 1,10-phenanthroline-5,6-epoxide (1.0 g, 5.1 mmol) in water (100 mL) was added to a 0.3 M aqueous solution of KCN (100 mL). The mixture was stirred at room temperature for 4 h. The white precipitated formed was filtered off, washed with a large amount of water, dried and recrystallized from methanol to afford pure (mass fraction purity > 0.999 %, measured by an Agilent 6890N GC apparatus equipped with a 30 m × 0.25 mm 5% phenylsilicone column) 5-cyano-1,10-phenanthroline (3.9 mmol, 76 %) as a white solid. ^{1}H NMR (300 MHz, $CDCl_3$) δ (ppm): 7.78 (m, 2H), 8.32 (m, 2H), 8.62 (d, 1H, J = 8.13 Hz), 9.30 (m, 2H). The ^{1}H NMR spectrum is consistent with that reported in the literature [10].

5-Nitro-1,10-phenanthroline was synthesized by reacting 1,10-phenanthroline with nitric acid in sulfuric acid according to a procedure reported in the literature [2]. Fuming nitric acid (15 mL) was added to a stirred solution of 1,10-phenanthroline (5 g, 27.8 mmol) in concentrated sulfuric acid at 160 °C. The mixture was stirred at 160 °C for 3 h and then poured into ice water. Saturated NaOH was then added until pH 3 was reached and a yellow solid precipitated. The precipitate was filtered off, washed with water and dried in vacuum. After recrystallization from ethanol, pure (mass fraction purity > 0.999 %, measured by an Agilent 6890N GC apparatus equipped with a 30 m × 0.25 mm 5% phenylsilicone column) 5-nitro-1,10-phenanthroline (5.9 g, 26.2 mmol, 94 %) was obtained as a yellow solid. ^{1}H NMR (300 MHz, $CDCl_3$) δ (ppm): 7.77 (m, 2H), 8.40 (dd, 1H, J = 8.1 Hz, J' = 1.7 Hz), 8.65 (s, 1H), 8.98 (dd, 1H, J = 8.6 Hz, J' = 1.6 Hz), 9.26 (dd, 1H, J = 4.3 Hz, J' = 1.6 Hz), 9.32 (dd, 1H, J = 4.4 Hz, J' = 1.7 Hz). The ^{1}H NMR spectrum is consistent with that reported in the literature [11].

5-Amino-1,10-phenanthroline was synthesized by reduction of 5-nitro-1,10-phenanthroline by hydrazine following a procedure previously reported in the literature [11]. 5-Nitro-1,10-phenanthroline (2.5 g, 11.1 mmol) was dissolved in absolute ethanol (50 mL) containing 10% Pd/C (0.5 g). After purging with Ar, hydrazine monohydrate (6.5 mL, 13.4 mmol) was added dropwise over a period of 30 minutes. The mixture was the stirred at 70 °C for 10 h. After cooling, the reaction mixture was filtrated and the filtrate was concentrated under reduced pressure. The yellow precipitate thus obtained was filtered, dried under vacuum and recrystallized from ethanol affording pure (mass fraction purity > 0.999 %, measured by an Agilent 6890N GC apparatus equipped with a 30 m × 0.25 mm 5% phenylsilicone column) 5-amino-1,10-phenanthroline (0.87 g, 4.46 mmol, 40 %) as a yellow solid. ^{1}H NMR (300 MHz, $CDCl_3$) δ (ppm): 3.58 (br, 2H), 6.94 (s, 1H), 7.50 (dd, 1H, J = 8.0 Hz, J' = 4.3 Hz), 7.64 (dd, 1H, J = 8.3 Hz, J' = 4.3 Hz), 7.98 (d, 1H, J = 7.3 Hz), 8.27 (d, 1H, J = 7.6 Hz), 8.94 (d, 1H, J = 3.2 Hz), 9.20 (d, 1H, J = 3.2 Hz). The ^{1}H NMR spectrum is consistent with that reported in the literature [11].
